# Alcohol related mental imagery: The effects of a priming dose in at risk drinkers^☆^^☆☆^

**DOI:** 10.1016/j.abrep.2017.08.003

**Published:** 2017-08-31

**Authors:** Michael Yates, Sunjeev K. Kamboj

**Affiliations:** a Research Department of Clinical Educational and Health Psychology, University College London, UK; b Clinical Psychopharmacology Unit, University College London, UK

**Keywords:** Mental imagery, Elaborated intrusion theory, Alcohol, Alcohol priming, Craving, Mind wandering

## Abstract

**Objectives:**

Drug related mental imagery is proposed to play a central role in addictive behaviour. However, little is known about such cognition or how it is pharmacologically modulated. Here, we test theoretical predictions of the ‘elaborated intrusion’ theory by comparing neutral with alcohol related mental imagery, and examine the effects of low dose alcohol on phenomenological aspects of this imagery.

**Methods:**

Alcohol related and neutral imagery was assessed after at risk drinkers (n = 40) consumed alcohol (0.3 g/kg) or placebo, in a crossover design. Sensory and visuospatial qualities of imagery, along with associated craving, positive affect and ‘mind wandering’ were assessed.

**Results:**

Alcohol related mental imagery was rated as more vivid and sensorially rich, effects that were larger following the priming dose of alcohol. In addition, mind wandering was substantially *lower* during alcohol versus neutral imagery, especially after alcohol consumption. First person perspective was more prevalent for alcohol imagery after alcohol, although the Drink × Imagery type interaction did not reach statistical significance. However, first person imagery was associated with higher levels of craving during alcohol related imagery.

**Conclusions:**

Alcohol related mental imagery differs phenomenologically from neutral imagery on a number of dimensions. Priming with alcohol may enable cognitive elaboration by biasing the output of controlled cognitive processing towards enhanced sensory elaboration and increased attention to alcohol related cognition. These feedforward effects may be involved in focusing cognitive and behavioural resources on alcohol acquisition/consumption through the elaboration and rehearsal of relevant goals and plans.

## Highlights

• Alcohol imagery was more vivid and sensorially rich than neutral imagery.
• Alcohol imagery was associated with greater pleasantness and craving.
• Mind-wandering was reduced during alcohol imagery.
• A priming dose (0.3 g/kg) of alcohol seemed to magnify these effects.

## 1 Introduction

Recent conceptualisations of disorders of reward suggest a critical role for visuospatial/sensory cognition in craving and drug use (Kavanagh, May, & Andrade, 2012). Sensory cues and internal physiological states are proposed to serve as inputs to cognitive processes that maintain and intensify craving through drug related intrusive imagery, which becomes increasingly elaborated through controlled cognitive processes (elaborated intrusion theory; May, Andrade, Panabokke, & Kavanagh, 2004). Elaboration serves as motivational driver, gearing behaviour towards acquisition of appetitive targets (May, Andrade, & Kavanagh, 2015). The elaborated intrusion (EI) theory provides the basis for a number of predictions in relation to the occurrence of intrusive desire related thoughts and controlled, elaboration processes (Kavanagh, Andrade, & May, 2005).

For example, craving is predicted to increase when there are a greater number of environmental and/or interoceptive cues available to assist the development of vivid appetitive imagery (May et al., 2004). Potent cues relevant to imagery elaboration include the interoceptive effects of the ingested drug (Paulus & Stewart, 2014), especially at low doses that do not interfere with the cognitive processes upon which mental imagery relies (e.g. working memory; Dry, Burns, Nettelbeck, Farquharson, & White, 2012). EI theory makes specific predictions in this regard: “More vivid imagery is likely when there are more environmental cues to assist its development, with the best set of cues provided by consuming a priming dose of the substance” (p. 449, Kavanagh et al., 2005). Given the proposed effects of drug related imagery on craving, a natural prediction is that drug priming should produce higher craving during drug related, versus neutral mental imagery. However, no study that we are aware of has examined such predictions experimentally using an acute drug dose.

Given the proposed role of mental imagery in motivating drug acquisition through the generation of drug related plans and goals (May et al., 2015) a reasonable additional prediction is that off target cognitive activity that interrupts elaboration would be less likely to (spontaneously) occur during drug related imagery compared to neutral imagery. Assessing the extent of off target mental activity (mind wandering) during drug related mental imagery may therefore be a method for gauging persistence or maintenance of elaborated drug related mental imagery, reflecting a narrowing of perceptual and cognitive activity (e.g. alcohol myopia; Steele & Josephs, 1990).

Studies of mental imagery in mood and anxiety disorders suggest that deliberate switching of imagery perspective from first to third person reduces emotional intensity in mood and anxiety disorders (Wallace-Hadrill & Kamboj, 2016). If this observation reflects a general relationship between visuospatial perspective and affect, egocentric perspective should be more prevalent and associated with higher levels of craving during drug related imagery in those for whom the drug has motivational salience.

Here we examine alcohol related and neutral mental imagery following a placebo drink and low dose alcohol in at risk drinkers. A low dose was used to instantiate a relevant physiological context to support imagery formation and elaboration (Kavanagh et al., 2005) without interfering with cognitive processes required for this imagery. A low dose also has the advantage of maximising concealment of treatment allocation (see blinding procedure below). Based on EI theory we predicted that alcohol imagery would be associated with higher levels of sensory features, greater vividness, craving and egocentricity, consistent with greater elaboration. Moreover, given that a priming dose of alcohol provides the interoceptive context for enhanced elaboration (Kavanagh et al., 2005), we predicted that alcohol consumption will be associated with more vivid, sensorially rich and egocentric alcohol imagery. We also examined mind wandering during neutral and alcohol imagery although given the exploratory nature of this assessment, no a priori directional hypotheses were made.

## 2 Materials and method

The study was approved by the University College London (UCL) Research Ethics Committee.

### 2.1 Participants

Participants were recruited based on risk of alcohol related harm through advertisements placed in the UCL campus and wider London area. Participants (n = 42) completed the first experimental session and n = 40 (20 men) returned for the second session and were included in the final analysis. Inclusion criteria were: age 18–65 years, fluency in English; scoring > 8 on the Alcohol Use Disorders Identification Test (AUDIT; Saunders, Aasland, Babor, De la Fuente, & Grant, 1993); exceeding UK government guidelines for alcohol consumption (2–3 units or 3–4 units/day for women and men respectively on ≥ 4 days/week; 1 unit = 8 g alcohol). In this sample, men consumed 21–25 units (n = 1), 26–30 units (n = 15) or, > 30 units (n = 4) and women consumed 14–20 units (n = 14), 21–25 units (n = 1), 26–30 units (n = 2) and > 30 units (n = 3) per week. Participants' ethnicities were: white (n = 30); nonwhite (n = 10). Exclusion criteria were: AUDIT scores indicating more severe alcohol problems (> 20), history of alcohol or other substance dependency or current/past treatment seeking for alcohol problems. Participants received £20 compensation.

### 2.2 Measures

Baseline craving was assessed using the 12-item Alcohol Craving Questionnaire-Short-Form (ACQ-SF; Singleton, Henningfield, & Tiffany, 1994) prior to the imagery procedure and interview on both sessions. Episodic craving during imagery was also assessed as part of the imagery assessment schedule using the ‘urge’ item (0 = no urge; 5 = extremely strong) from the Mood and Physical Symptom Scale (MPSS; West & Hajek, 2004).

Drinking motives were assessed using the Drinking Motives Questionnaire (DMQ-R; Cooper, 1994) to quantify maladaptive enhancement and coping, as well as social and conformity motives.

The Spontaneous Use of Imagery Scale (SUIS; Reisberg, Pearson, & Kosslyn, 2003) assessed mental imagery in everyday life. Additionally, the Positive and Negative Affect Schedule (PANAS; Watson & Clark, 1999) was used to assess stability of mood (previous week) on both sessions.

### 2.3 Mental imagery procedure

The mental imagery procedure consisted of two phases: i) a description of, and prompts to generate imagery and ii) an assessment schedule that tapped various phenomenological and subjective aspects of the elicited imagery (Winfield & Kamboj, 2010). Participants first listened to a standardised audio recorded description of the nature of mental imagery, emphasising its multisensory nature. Participants were then given a neutral or alcohol related verbal prompt, with order randomised and balanced across session. The alternative prompt was given immediately after assessment of imagery from the first prompt.

Alcohol and neutral imagery prompts respectively were: “*imagine drinking your favourite alcoholic drink*” and “*imagine watching your favourite television programme*” (adapted from Tiggemann & Kemps, 2005). Participants closed their eyes to indicate when they started the imagery task. After 1 min, they provided responses to items on the imagery assessment schedule (~ 5-min per prompt) in the order listed below after each imagery condition.

*Mind wandering*. This was described as a tendency to spontaneously engage in task irrelevant mentation. This was rated on a 0 = not at all to 10 = continuously. As in other mind wandering research, no probes were used to assess content of task unrelated thoughts.

*Imagery suppression* was described as a deliberate attempt to disengage from imagery (0–10 scale). Since participants scored at floor levels on suppression (*Mean* = 1.28, *SD* = 1.81) this data will not be discussed further.

*Vividness* related to “*clarity*, *crispness*, *sense of* ‘*realness*’ *of the image*” and was rated on a 0 = not at all to 10 = extremely vivid scale.

*Pleasantness/unpleasantness* was also rated on a 11-point scale: − 5 = *very unpleasant*; + 5 = *very pleasant*; 0 = *neutral*.

*Sensory features*. Participants indicted the presence of visual, auditory, olfactory, and taste features, each scoring ‘1’ (range: 0–4).

*First/third person perspectives* was assessed as in similar studies (see Wallace-Hadrill & Kamboj, 2016) and treated dichotomously.

*Previous experience of imagery*, *prospection* versus *memory and dynamism*: Participants indicated whether the image had been (i) experienced previously outside of the experiment (old versus novel), (ii) a memory or a future scenario (iii) dynamic versus static, all rated on binary scales. However, for the prospection versus memory item, there was a significant amount of ‘missing data’ (≤ 17.5% across conditions) because participants were unable to categorise imagery as a future event or memory. The remaining responses were primarily future events across conditions (80–94%). Given overall ceiling levels and frequency of nonresponding, analyses of this data was not justified and is not discussed further. Similarly, dynamism was not analysed due to limited variability between conditions (75–80% of imagery was rated as dynamic across conditions).

### 2.4 Beverage preparation and administration

Various volumes of 37.5% vodka - depending participant weight - were diluted with tonic to achieve a final volume of 500 ml at 0.3 g/kg. This was divided into three ~ 166 ml portions, each containing four drops of Tabasco as a masking agent. Placebo contained only tonic and Tabasco, with vodka mist sprayed around, and 2–3 drops added to, the placebo containing cup to improve condition concealment. Participants consumed each portion within 5-min, and only drank the next portion after a full 5-min had elapsed. After the 15-min consumption period, there was a passive (reading) period of 15-min to allow absorption. Although peak BAC levels may not be achieved within 15-min after the end of drinking (30-min after the drinking procedure commenced), it is expected that low dose alcohol is absorbed and exerts stable psychophysiological effects by 15-min in heavy drinkers (e.g. King & Byars, 2004). The same interval (15-min + 15-min) was used on the placebo session.

### 2.5 Design and procedure

A factorial, repeated measures, within subjects design was used with ‘Drink condition’ (alcohol/placebo) and ‘Imagery type’ (alcohol/neutral) as within subjects factors. In each of two sessions (placebo and 0.3 g/kg alcohol), participants completed the imagery task for the alcohol or neutral scenario, followed by the other scenario, with order counterbalanced over participants across sessions.

Assessment of hazardous/harmful drinking (AUDIT; units/week) occurred at screening. Participants attended two experimental sessions, refraining from alcohol (≥ 8 h), recreational drugs, sedatives and antihistamines (≥ 24 h), caffeine (≥ 3 h) and high calorie foods (≥ 2 h) prior to sessions. They were informed that they would receive alcohol on one occasion and a similar nonalcoholic drink on the alternative session. Testing commenced 4 pm–7 pm. Upon arrival, a breathalyser (Lion Instruments, UK) reading of 0.00 was required to confirm absence of blood alcohol (all participants provided this). No breathalyser readings were taken after alcohol administration. Written informed consent, participant weight, estimated weekly alcohol consumption and alcohol use history (period of consumption at the current level; age at regular drinking) were obtained on session one. Participants also completed the DMQ-R, SUIS and PANAS measures prior to alcohol/placebo consumption on session one.

Drinks were prepared in an adjoining lab and were presented in three identical cups (see Beverage Preparation section above). After consumption and an absorption period, participants completed the imagery procedure, which apart from order of imagery cue words (neutral/alcohol) was identical on both sessions. Blinding was assessed at the end of each session (“*Do you think the drink you received contained alcohol?*”).

### 2.6 Statistical analysis

*Means* ± *SD* are reported unless otherwise indicated. Continuous variables were analysed (SPSS, version 22) using mixed factorial ANOVA with imagery type (neutral, alcohol related) × Drink condition (placebo, alcohol) as factors. Repeated categorical variables (perspective and previous experience) were analysed using generalised estimating equations. Effects of Drink and Imagery order were investigated using 3-way ANOVAs, which showed no order effects (F values < 1).

Post hoc tests of significant interactions were Bonferroni corrected. Reporting of specific individual effect sizes (Cohen's *d*; adjusted for within subjects correlations between levels of the dependent variable) was based a priori hypotheses (outlined in the introduction). All reported *p* values are two tailed.

## 3 Results

Participant characteristics are outlined in Table 1. The pattern of drinking motive scores was consistent with at risk social drinkers. ACQ and PANAS scores were stable across the experimental sessions (*t* < 1).

**Table 1:** Participant characteristics and drinking behaviours.

	Mean (± SD)
Age (years)	28.08 (8.63)
Consuming at current level (months)	42.98 (30.17)
Age at regular drinking	15.42 (1.50)
Number drinking days/perweek	4.05 (1.11)
AUDIT	14.3 (4.97)
Drinking motives	
Social	19.43 (3.44)
Enhancement	15.88 (3.90)
Coping	11.58 (4.19)
Conformity	8.05 (3.01)

### 3.1 Guess on treatment

On the alcohol session, 47.5% of participants correctly identified alcohol content. On the placebo session, 54.5% incorrectly guessed that the drink containing alcohol. Chance level accuracy suggests effective concealment of the low alcohol dose.

### 3.2 Effects of alcohol on phenomenological characteristics of alcohol related and neutral mental imagery

#### 3.2.1 Sensory and affective qualities of alcohol related imagery

Imagery vividness was higher after alcohol than placebo (*F*(1,39) = 6.86, *p* = 0.013, *η*_*p*_^*2*^ = 0.15) and vividness of alcohol imagery was higher than neutral imagery (*F*(1,39) = 83.64, *p* < 0.001, *η*_*p*_^*2*^ = 0.68) but there was no interaction involving drink condition (*F*(1, 39) = 1.079, *p* > 0.1; Table 2). The effect sizes for vividness of alcohol related imagery were *d* = 1.27 and *d* = 0.87 after 0.3 g/kg alcohol and placebo respectively.

**Table 2:** Sensory, affective (*Mean* ± *SD*) and perspective and novelty (n and %) characteristics according to Drink condition and Imagery type.

Sensory/affective	Drink condition			
	Placebo		Alcohol	
	Neutral imagery	Alcohol related	Neutral imagery	Alcohol related
Vividness^a^, ^b^	5.58 (1.89)	7.10 (1.50)	6.03 (1.70)	7.90 (1.15)
Sensory features^a^, ^b^	2.23 (1.03)	2.78 (1.12)	2.45 (0.81)	3.30 (0.72)
Pleasantness^b^	2.08 (1.80)	2.83 (1.41)	2.25 (1.78)	3.05 (1.43)
Craving^a^, ^b^	1.58 (0.96)	2.70 (1.24)	1.78 (0.97)	3.23 (1.21)

See text for details on item scoring.

a Main effect of drink condition.

b Main effect of image type.

There were also more sensory features across levels of Imagery type after alcohol (F(1,39) = 10.29, *p* = 0.003, *η*_*p*_^*2*^ = 0.20). In addition, Table 2 shows a larger number of sensory features for alcohol versus neutral imagery across Drink conditions (*F*(1,39) = 34.90, *p* < 0.001, *η*_*p*_^*2*^ = 0.472). While the Drink × Imagery type interaction was not significant (*F*(1,39) = 2.58, *p* = 0.116, *η*_*p*_^*2*^ = 0.062) it is again noteworthy that the sensory features advantage for alcohol imagery over neutral imagery was (two fold) larger after alcohol (*d* = 1.10) than placebo (*d* = 0.51).

In relation to affective qualities (Table 2), there was only a main effect of Imagery type, indicative of greater pleasantness of alcohol imagery (*F*(1,39) = 12.93, *p* = 0.001, *η*_*p*_^*2*^ = 0.25; effects involving Drink type: *F* values < 1). However craving was higher after alcohol across levels of Imagery type (*F*(1,39) = 5.40, *p* = 0.025, *η*_*p*_^*2*^ = 0.12) and in response to alcohol imagery across levels of Drink (*F*(1,39) = 76.18, *p* < 0.001, *η*_*p*_^*2*^ = 0.66; Table 2), although their interaction was not significant (*F*(1,39) = 2.90, *p* = 0.096, *η*_*p*_^*2*^ = 0.07). Large effects on craving during alcohol relative to neutral imagery were found after placebo (*d* = 1.00) and alcohol (*d* = 1.30).

#### 3.2.2 Imagery perspective

Similar proportions of first and third person perspective were reported after placebo and alcohol (*B* = 0.40, *SE* = 0.28, *p* > 0.1). The effect of Imagery type was at trend level (*B* = 0.61, *SE* = 0.34, *p* = 0.076), as was the Imagery type × Drink interaction (*B* = 1.24, *SE* = 0.67, *p* > 0.064). The latter trend appeared to reflect a greater proportion of first person alcohol imagery after alcohol consumption (Table 3), which was considerably higher (87.5%) than all other conditions (≤ 57.5%).

**Table 3:** Frequency (n and %) of first versus third person perspective (top half) and whether the imagery evoked during the imagery task had previously been experienced or was novel.

	Drink condition			
	Placebo		Alcohol	
	Neutral imagery	Alcohol related	Neutral imagery	Alcohol related
Perspective				
First person	17 (42.5)	23 (57.5)	21 (52.5)	35 (87.5)
Third person	23 (57.5)	17 (42.5)	19 (47.5)	5 (12.5)
Novelty^a^				
Previously experienced	16 (40)	31 (77.5)	19 (47.5)	28 (70)
Novel	24 (60)	9 (22.5)	21 (52.5)	12 (30)

a Main effect of Image type.

To specifically test the idea that first person perspective is associated with greater reward/desire-related affect (Wallace-Hadrill & Kamboj, 2016) we compared craving in participants who predominantly experienced first versus third person perspectives during alcohol imagery and during neutral imagery (this analysis was restricted to the placebo condition because of the unbalanced sample sizes in the alcohol drink condition). Craving levels were higher in participants with first person perspective during alcohol imagery (*t*(38) = 2.75, *p* = 0.009, *d* = 0.94) whereas craving did not differ between participants with first with third person perspectives for neutral images (t values < 1).

#### 3.2.3 Previous experience of evoked mental imagery

There was a main effect of Imagery type (*B* = 0.95, SE = 0.42, *p* = 0.025) indicative of greater previous experience of the alcohol image compared to the neutral image (Table 3), but not of Drink condition (*B* = 0.39, SE = 0.47, *p* *>* 0.1), and no 3-way interaction (*B* = 0.70, SE = 0.57, *p* *>* 0.1).

#### 3.2.4 Mind wandering

Main effects of Drink condition (*F*(1,39) = 41.00, *p* < 0.001, *η*_*p*_^*2*^ = 0.51) and Imagery type (*F*(1,39) = 88.12, *p* < 0.001, *η*_*p*_^*2*^ = 0.69) were qualified by their interaction (*F* (1, 39) = 10.70, *p* = 0.002, *η*_*p*_^*2*^ = 0.22) on mind wandering. Bonferroni corrected pairwise comparisons indicated that in the placebo condition there was significantly less mind wandering during alcohol versus neutral imagery (*p* < 0.001; *d* = 0.74).

**Fig. 1:**
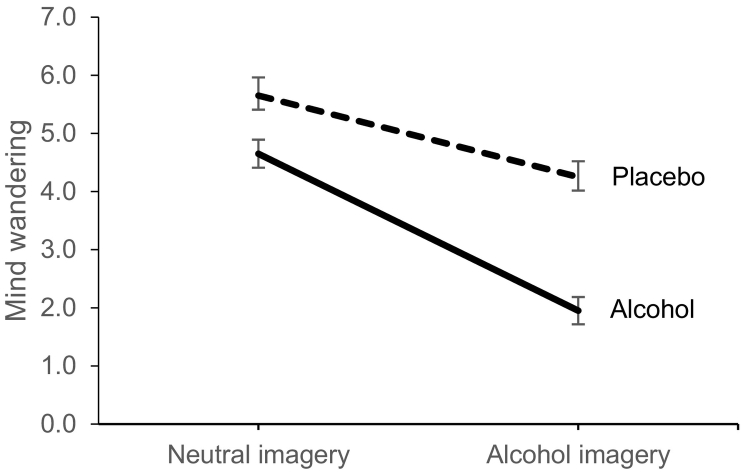
Mean (± SEM) scores for mind wandering retrospectively rated immediately after neutral and alcohol related mental imagery after placebo (dashed line) and alcohol (solid black line).

The difference in mind wandering after alcohol consumption during alcohol, compared to neutral imagery, was considerably larger (*p* < 0.001; *d* = 1.76; Fig. 1). In relation to the effects of alcohol, it is clear (Fig. 1) that a priming dose was associated with a generalised reduction in mind wandering, although for neutral imagery, this reduction (compared to placebo) was moderate (*p* = 0.007, *d* = 0.56), whereas for alcohol imagery the effect size of this reduction was > 2.5 times larger (*p* < 0.001, *d* = 1.44).

#### 3.2.5 Effects on imagery outcomes depending on correct guessing of treatment allocation

Since correct guessing of treatment condition could have introduced expectancy effects, the above analyses were repeated with ‘guess on treatment’ (correct/incorrect) on each of the two sessions as an additional (between subjects) factor. These showed no 3-way interactions involving guess on treatment (*p* > 0.1) and the pattern of significant main effects and the 2-way interaction (mind wandering) were retained in these analyses.

## 4 Discussion

In this study, we examine the effects of alcohol on the phenomenology of alcohol related mental imagery. Our primary findings were that the expected indices of sensory/visuospatial elaboration (vividness and number of sensory features) and affective responding (craving, pleasantness) were higher during alcohol related than neutral imagery. Although a number of interactions were not significant, descriptive effects were in the predicted direction. In particular, effects on vividness, and especially, sensory features, were (1.5 to 2-fold) larger for alcohol relative to neutral imagery following alcohol consumption, and in line with predictions outlined in the introduction.

We also observed a novel effect of a priming dose of alcohol on mind wandering in the context of alcohol mental imagery. Finally, alcohol related imagery appeared more frequently to be egocentric following alcohol (87.5% versus ≤ 57.5%) and egocentric alcohol imagery was associated with greater craving.

These findings are broadly consistent with the elaborated intrusion (EI) theory (Kavanagh et al., 2005). They complement previous cross sectional empirical work which has tended to explore the relationship between sensory elaboration, craving, patterns of drinking, instances of involuntary alcohol related imagery using similar indices to those used here or a questionnaire measure based on the EI theory (Connor et al., 2014, Kavanagh et al., 2009, Statham et al., 2011). However, this is the first *experimental pharmacological* study of drug related imagery of which we are aware.

Our preliminary findings on mind wandering are intriguing given that drug relevant physiological states (intoxication or withdrawal) have previously been shown to *increase* mind wandering (Sayette et al., 2009, Sayette et al., 2010). However, these previous findings related to a nondrug relevant activity (reading; Sayette et al., 2009, Sayette et al., 2010). Moreover, they were found for non ‘self-caught’ mind wandering (i.e. mind wandering without awareness) after alcohol (Sayette et al., 2009), although subjective ‘task disengagement’ has also been noted in related studies following alcohol (Finnigan, Schulze, & Smallwood, 2007). It is important to note however, that the dose used here (0.3 g/kg) was much smaller than used in these previous studies (≥ 0.8 g/kg). It is also unclear how a small dose would affect mind wandering in an experimental paradigm that involved *continuous* self-monitoring, and probing for task disengagement (assaying meta-awareness), as used by Sayette et al. (2009), rather than retrospective reporting. Given the methodological limitations of our mind wandering measure, our findings, while intriguing, should repeated with alternative methodologies. Nonetheless, preliminarily, they are supportive of EI theory in terms of a focusing of cognitive resources on mental imagery, which could support elaboration.

We acknowledge a number of limitations of the present study, which imply directions for future research on drug effects on mental imagery. Firstly, we used a single (participant) blind design, and as such, there is scope for bias associated with nonblinding of experimenter. Secondly, our focus was on controlled (nonautomatic) features of alcohol related imagery, which addresses the ‘elaboration’ aspect of EI theory, rather than the ‘intrusion’ (automatic) aspect. It is possible that the elaboration processes following intrusive desire related mental imagery are qualitatively different to voluntary imagery. To the extent that intrusive desire related imagery enters conscious awareness, such imagery could be subjected to similar phenomenological examination, although this would likely need to occur in naturalistic settings (e.g. Kavanagh et al., 2009).

The absence of a control condition involving nonalcohol consummatory imagery did not allow us to distinguish specific effects of (alcohol on) alcohol imagery versus general reward related imagery. It may be, for example, that frequent rehearsal and a rich episodic memory base upon which to draw during alcohol imagery resulted in enhanced vividness and sensory detail and that similar effects would be observed for imagery for food or other rewarding activity. In other words, our observations may be secondary to learning history and memory (indeed, rehearsal, or ‘past experience’ of imagery seemed more common for alcohol versus neutral imagery). In addition, the neutral condition used here may have inherently biased certain observations. For example, imagery of a passive activity, such as watching television, may have encouraged a third person perspective (e.g. imagery of oneself watching television). However, the limited difference in imagery perspective between alcohol and neutral imagery in the placebo conditions is not consistent with this idea. Nor does it explain a potential (though nonsignificant) bias towards first person alcohol imagery following alcohol consumption. Nonetheless, future studies should consider alternative appetitive control imagery conditions, and compare at risk drinkers with social/infrequent drinkers (for whom alcohol has lower incentive value, and may therefore show a different pattern of effects to those observed here). Alternative, neutral imagery involving ‘consumption’, but no reward associations (e.g. brushing one's teeth), would also have provided an alternative control condition. Finally, it should be noted that the suggestive, but nonsignificant effects on perspective may reflect a lack of power to detect binary differences in first and third person imagery.

## 5 Conclusions

To summarise and conclude, this study describes novel aspects of reward related imagery and its modification by alcohol. These findings are broadly consistent with predictions from EI theory and suggest some novel areas of further research.

## Role of funding source

The study was funded by UCL. The institution had no role in preparation of the publication or the decision to publish.

## Contributors

SKK designed the study, conducted the analyses and wrote the paper. MY contributed to design and collected all data. Both authors have approved the final article.

## Conflicts of interests

The authors confirm that they have no conflicts of interest.
